# Identifying priority technical and context-specific issues in improving the conduct, reporting and use of health economic evaluation in low- and middle-income countries

**DOI:** 10.1186/s12961-018-0280-6

**Published:** 2018-02-05

**Authors:** Alia Luz, Benjarin Santatiwongchai, Juntana Pattanaphesaj, Yot Teerawattananon

**Affiliations:** Health Intervention and Technology Assessment Program (HITAP), Ministry of Health, Nonthaburi, Thailand

## Abstract

**Background:**

The use of economic evaluation in healthcare policies and decision-making, which is limited in low- and middle-income countries (LMICs), might be promoted through the improvement of the conduct and reporting of studies. Although the literature indicates that there are many issues affecting the conduct, reporting and use of this evidence, it is unclear which factors should be prioritised in finding solutions. This study aims to identify the top priority issues that impede the conduct, reporting and use of economic evaluation as well as potential solutions as an input for future research topics by the international Decision Support Initiative and other movements.

**Methods:**

A survey on issues regarding the conduct, reporting and use of economic evaluation as well as on potential solutions was conducted using an online questionnaire among researchers who have experience in conducting economic evaluations in LMICs. The respondents were requested to consider the list of issues provided, rank the most important ones and propose solutions. A scoring system was applied to derive the ranking of difficulties according to researchers’ responses. Issues were grouped into technical and context-specific difficulties and analysed separately as a whole and by region.

**Results:**

Researchers considered the lack of quality local clinical data, poor reporting and insufficient data to conduct the analysis from the chosen perspective as the most important technical difficulties. On the other hand, the non-integration of economic evaluations into decision-making was considered the most important context-specific issue. Finally, context-specific issues were considered the larger barrier to the use of economic evaluation.

**Conclusion:**

The technical issues that were considered most important were closely linked with the lack of an appropriately functioning information system as well as the capacity to generate essential contextual information (e.g. data and locally relevant utility values), especially when the methodology is complex. To overcome this, simpler approaches to collect data that yields information of comparable quality to more rigorous methods should be developed. The international community can play a major role through research on methodologies feasible for LMIC settings as well as in building research capacity in countries. Context-specific issues, which were recognised as larger barriers, should be improved in parallel.

**Electronic supplementary material:**

The online version of this article (10.1186/s12961-018-0280-6) contains supplementary material, which is available to authorized users.

## Background

Economic evaluation is a process that identifies, measures, valuates and compares the costs and consequences of at least two alternative courses of action. It aims to determine the efficiency or cost-effectiveness of the implementation of studied alternatives [1]. The use of this information to inform priority-setting and resource allocation is more common in high-income countries (HICs), such as the United Kingdom, Canada, Australia, New Zealand, etc., where this field of research is more established. On the other hand, the use of economic evaluation evidence in policy decision-making in low- and middle-income countries (LMICs) is frequently found difficult due to the range of barriers and concerns encountered throughout the process. These are rooted in both evidence generation and evidence utilisation, and are caused by both technical difficulties and non-technical limitations, which are discussed in a number of economic evaluations, reviews of economic evaluation or normative papers [2–5].

Evaluation practitioners in LMICs often face capacity and technical challenges in the conduct of economic evaluation compared to those in HICs. This is evident in a review comparing the methodology of economic evaluation in HICs and LMICs, which reveals major differences in the methodology and indicates that there could be many limitations that hinder researchers conducting studies in LMICs using practices used in HICs [6]. Since economic evaluation is a field of research that relies heavily on the data used and involves judgement and assumptions, economic evaluation practitioners need to carefully consider the quality of their methodology and inputs used for the analysis. Mistakes may significantly compromise the usefulness of the findings and impede their policy usability and application [5, 7]. However, experienced economic evaluation practitioners are scarce in the LMIC context, and even when there are experts, the availability of quality essential inputs in the local context are often limited.

Findings in many studies have further indicated that the issues on availability and quality of data used in the economic evaluation as well as relevant research standards are significant in LMICs [5, 6, 8]. Although some data, such as treatment effect, is considered transferable across settings [9] (and therefore researchers may choose to borrow from other contexts), application of this approach to other types of inputs that are more context-specific (e.g. epidemiological, cost and quality of life data) is much less feasible. An alternative would be to extrapolate the inputs from the best available data, which is not necessarily of high quality. The application of these approaches without careful consideration will limit the relevance of inputs used in the analysis to the study context and subsequently compromise its potential use in policy-making [5]. Moreover, there is usually a lack of consensus on standard guidelines for both methodology and reporting of economic evaluation in LMICs [6]. In addition to shortcomings in terms of quality of studies, this results in high methodological and reporting variations and therefore limited comparability across studies in the same context, which poses a difficulty in considering them in policy-making. The results of this are reflected in evidence which found that there are variations in the quality of studies in LMICs. For example, though non-specific to health economics, a World Bank review of projects under their umbrella showed that there are many misconceptions in the conduct and components of economic evaluation [10].

Solving these technical shortfalls will promote the use of economic evaluation in policy decision-making, but careful consideration should also be paid to non-technical issues. It should be noted that, although the likelihood of high quality and context-relevant economic evaluations being used is higher, these improvements cannot guarantee more and better use of the evidence. This can be viewed as the result of an outstanding example of a non-technical barrier, namely the lack of formal mechanisms to consider the evidence for decision-making. The availability of quality evidence will not be meaningful unless policy-makers acknowledge its existence and usefulness, and unless there is a capacity and supportive mechanism to translate it into policy. On the other hand, strong political buy-in will lead to a demand and financial support for quality economic evaluations and political support to develop the systems and infrastructure needed for the conduct, and eventually the use, of quality economic evaluation. These issues are overarching concerns that need to be tackled simultaneously with technical issues to achieve success in integrating economic evaluations into policy-making.

With a broad range of issues to be solved, prioritisation is needed to identify the most important challenges. Although there have been attempts to identify these challenges, the majority of the research is disease-specific, country-specific or without a focus on LMICs [5–27]. Moreover, among the various existing issues in LMICs, it has never been made explicit which of the issues are perceived to be of greater priority. This study therefore aims to identify the priority issues that impede the conduct, reporting and use of economic evaluation in LMICs and to explore potential solutions for these priority issues.

For the purpose of this study, issues were categorised into technical issues and context-specific issues. Technical issues are those that are directly linked to the feature, methodology and reporting of economic evaluation and can be solved through changes in the methods or methodological reporting specifications. It is worth noting that some technical issues are limitations that researchers face in the conduct of economic evaluation, while others are weaknesses in the studies produced due to inadequate research capacity. Therefore, technical issues can be sub-grouped into lack of data, an inappropriate use of data, lack of commonly accepted methods, and an inappropriate use of methods. On the other hand, context-specific issues are defined as non-methodological issues that are bound to the situation in the researchers’ context and cannot be solved with the adjustment or standardisation of methodology. At their foundation, context-specific issues centre around a lack of capacity and resources in evidence users, evidence generators and relevant supportive mechanisms in the context. While technical issues are focused on evidence generation and use, context-specific issues focus on the effective application and use of the studies produced. It is important to highlight that these issues are closely intertwined – one context-specific issue may affect another as well as context-specific issues affecting technical issues and vice versa.

In an attempt to build upon real-world experience of those who have conducted the studies and have been hard-pressed with those challenges, economic evaluation practitioners in LMICs are the sources of information since they are in the best position to provide a perspective on the technical details that evidence-users may not be familiar with. A survey approach was adopted since this study aims to explore opinions that cannot be gathered through literature reviews. This study asks the economic evaluation practitioners to assess the technical and context-specific issues they have encountered in their career with the objective of selecting priority technical issues and context-specific issues as well as comparing which barrier has more impact on the conduct and use of economic evaluation evidence.

The findings of this study will be an input to the International Decision Support Initiative (iDSI), through which this study is initiated, and potentially for the future research of other global donors seeking to provide solutions for these issues.

## Methods

### Study design

This study was conducted as a survey using a web-based online questionnaire. The questionnaire requests the respondent to consider the list of technical and context-specific issues provided and then to rank the those that they considered most important in their contexts (Question used for technical issues: “Please answer the following questions regarding the methodology for economic evaluations. The following are commonly met technical weaknesses that can hamper the quality and the use of economic evaluations in LMICs”. Question used for context-specific issues: “What other non-technical contextual factors affect the effective application and use of economic evaluation in LMICs?”). The respondents could choose to propose important issues not yet in the list provided (a maximum of one issue for each category). Moreover, respondents could propose solutions that they consider relevant to the issues chosen. The questionnaire went through consultation with experts to ensure face validity and was piloted before the conduct of the online survey. This survey was conducted anonymously and did not collect sensitive information of the respondents nor did it affect the respondents physically or psychologically, and the data was not publicised at individual level. Therefore, ethical approval was not applicable nor sought.

### Constructing the list of potential issues

Potential issues which might affect the methodological quality of economic evaluations were identified from existing key studies that discuss challenges, barriers or flaws in the conduct and quality of economic evaluations [2–4, 11–31], as well as primary reviews to identify methodological shortcomings of economic evaluation studies as appraised in Centre for Reviews and Dissemination (CRD) critical commentaries. Although a systematic review was not conducted since the study did not seek to derive a conclusion, but rather to guide the development of the list which respondents further considered and contributed to, the review was extensive enough to enhance the representativeness of the review results. Twenty-five studies discussing challenges, barriers and flaws in the conduct of economic evaluation in LMICs were identified from the MEDLINE (PubMed), World Bank, and WHO databases. Moreover, a review of individual economic evaluations was also conducted through the National Health System Economic Evaluation Database (NHS EED) hosted by the CRD, University of York, United Kingdom. NHS EED is an online database that archives studies meeting the criteria for economic evaluations from different databases, e.g. MEDLINE and EMBASE, and provides critical abstracts that appraise the methodology of the studies. In this review, a search using names of LMICs returned 180 studies with critical abstracts conducted in LMICs. Of these, 100 were randomly selected to extract methodological problems as appraised by the CRD reviewers. The full list of issues identified through both channels, along with the frequency of the issues being mentioned, can be found in Tables 1 and 2.

**Table 1:** Frequency of issues being mentioned in included key publications from PubMed (n = 25)

Issues	Number of studies in which the issue is mentioned
Technical issues	
Poor reporting	9
Perspective not stated	7
Methodology not presented in a clear and reproducible manner	2
Disaggregated result not presented	1
Funding sources not reported	1
Ethical issues not discussed	1
Lack of high-quality local clinical data	7
Lack of local utility data	4
Sensitivity analysis not properly characterised	4
Some relevant cost data omitted	3
Incremental analysis not performed	3
Clinical data not based on systematic review	2
Lack of reliable cost data	2
Discounting not performed, if relevant	2
Methodology lacks standard, transparent methods	2
Comparator not appropriate	1
Variations among costs, effects and cost-effectiveness data within and between settings	1
No objective budget constraints or threshold applied	1
No reference case specific to developing contexts	1
Economic evaluation is not included in a formal process to support decision-making process	1
Limited local research capacity	1
Limited local good quality journal with a high standard process of review	1
Misunderstanding between researchers, academia and policy-makers	1

**Table 2:** Issues in selected studies from the Centre for Reviews and Dissemination database (*n* = 100)

Issues	Numbers of studies in which the issue is mentioned
Poor reporting	81
Perspective of analysis not stated	37
Price year not reported	37
Decision model not described, if relevant	21
Limited details on utility/disutility data	15
Source of cost data not provided	12
Discount rate for cost not provided	12
Limited details on source of effectiveness data	11
Limited details on disaggregated cost data	11
Sources of effectiveness data not provided	7
Not clear whether all relevant options were included	5
Details on study population not provided	5
Justification of the comparator was not provided	4
The comparator was unclear	3
Details of comparators were not provided	2
Unclear whether discounting is performed for effectiveness	2
Discount rate for effectiveness not provided	2
Details on intervention is not provided	1
Unclear whether discounting is performed for cost	1
Limited details on currency conversion	1
No specific threshold applied	78
Incremental analysis not performed	41
Sensitivity analysis not performed	31
Health measures used not appropriate	28
All relevant evidence not included	17
Discounting of cost not appropriately performed	16
Sources of effectiveness should be improved	11
Some relevant costs are omitted	7
Charges used instead of cost	4
Sources of cost data should be improved	3
Discounting of effectiveness not appropriately performed	2

The issues identified from the review were summarised and categorised into either technical or context-specific issues. Triangulated with inputs from experts, lists of technical issues and context-specific issues were constructed. Details of issues included in the questionnaire can be found in Additional file 1.

### Study population

The study population comprised researchers who had completed at least one economic evaluation project as the primary investigator or as a part of a team in LMICs, defined per World Data Bank classifications as of 2015. In addition to the main survey component, qualifier questions, whether the respondent has experience in conducting an economic evaluation in LMICs and whether the respondent gives consent for the use of their response for analysis and publication, were also included. If respondents chose ‘no’ for any of the qualifier questions, the survey ended without proceeding further.

Potential respondents were contacted through research networks and individually. For the health technology assessment (HTA) networks, invitation to participate in the survey was sent to the secretariat of health economic and outcome research networks as well as various health technology assessment networks in different regions, who distributed the survey to their network members in the mailing list. The participating networks included the African Health Economics and Policy Association, HTA Network of the Americas and HTAsiaLink. Individuals who were contacted were the corresponding authors of economic evaluations conducted in LMICs in NHS EED. A search using the names of all LMICs returned 568 hits, of which 180 studies were conducted in LMICs; for these, the corresponding authors were contacted through the e-mail address provided.

### Ranking and data analysis

Since the number of technical issues identified was significantly larger than context-specific issues, respondents were asked to rank the three most important technical issues along with the most important context-specific issue. The solutions proposed that fell within the same concerns or areas were grouped together. The results of the ranked issues were then analysed as a whole and by WHO regions [32], considered by the region that respondents work in.

The top priority issues for technical and context-specific issues were analysed separately using a scoring system. For each response, the technical issue that was ranked first, second and third were assigned the score of 3, 2, and 1, respectively, while the context-specific issue that was picked got the score of 3. Thus, higher scores indicate a higher priority. The scores for each issue were summarised across responses and ordered by scores to derive the list of top priority issues. Where there were two or more issues that received the same score, the frequencies of the issues being ranked in the first, second, and third levels were considered.

## Results

In total, there were 927 recruited respondents. Of these, 178 people responded to the invitation (19% response rate). However, only 110 passed the qualifier questions. The respondent characteristics can be found in Table 3. Further characteristics of respondents can be found in Additional file 2.

**Table 3:** Characteristics of respondents

Characteristics	Number and percentage of respondents
Highest level of education completed (*n* = 110)	
Undergraduate	11 (10%)
Masters	58 (53%)
Doctorate	41 (35%)
Economic evaluation as a major part of the respondent study (*n* = 110)	
Yes	70 (64%)
No	40 (36%)
Years of experience in the field of economic evaluation (*n* = 105)	
0–5	49 (47%)
6–10	32 (30%)
11–15	8 (8%)
16–20	8 (8%)
21–25	2 (2%)
26 or more	5 (5%)
Affiliation (*n* = 110, multiple answers allowed)	
Academic	46 (42%)
Public health institute	46 (42%)
Governmental research bodies	7 (6%)
Ministry of Health	7 (6%)
Governmental bodies (unspecified)	4 (4%)
Consultancies	4 (4%)
Industries	4 (4%)
Others	4 (4%)
Regions of affiliation (*n* = 110, multiple answers allowed)	
Africa	34 (32%)
America	18 (17%)
Eastern Mediterranean	6 (6%)
Europe	16 (15%)
South East Asia	42 (40%)
Western Pacific	34 (32%)

### Issues

#### Priority technical issues

For technical issues, the top five priority issues were the lack of essential local clinical data, poor reporting, insufficient data to conduct the analysis from the chosen perspective, lack of a standard practice that is relevant to LMICs, and the lack of a tool to derive quality-adjusted life years (QALYs) and disability-adjusted life years (DALYs). The score and frequencies for each issue are presented in Table 4.

**Table 4:** Ranked technical issues, presented as groups of related issues and by rank

Priority rank	Technical issue	Score	Frequency in first rank
Lack of relevant data			
1	Lack of high-quality local clinical data, where such data are critical to the decision	80	21%
3	Insufficient data to conduct study from chosen perspective	57	9%
5	Absence of locally relevant health state preference data suitable for estimating QALYs or DALYs	43	7%
Lack of commonly accepted standard or methods			
2	Poor reporting	67	21%
4	A lack of commonly accepted standards for economic evaluation that is relevant to the LMIC for which the analysis is undertaken	57	19%
Inappropriate use of methods			
6	Inappropriate choice of comparator(s)	29	7%
7	No budget constraints or thresholds considered	26	5%
8	Generalisability not discussed	14	3%
10	Equity and/or gender implications not considered	12	0%
11	No incremental analysis	11	1%
12	No, or inappropriate, sensitivity analysis	10	0%
13	All impacts implied by the chosen perspective not investigated	10	3%
15	Time horizon too short to capture relevant costs and health effects	9	1%
Inappropriate use of data			
9	Clinical data not based on systematic review or primary clinical data not compared with similar studies done elsewhere	12	1%
14	Uncritical use of charges for cost data	9	1%

**Fig. 1:**
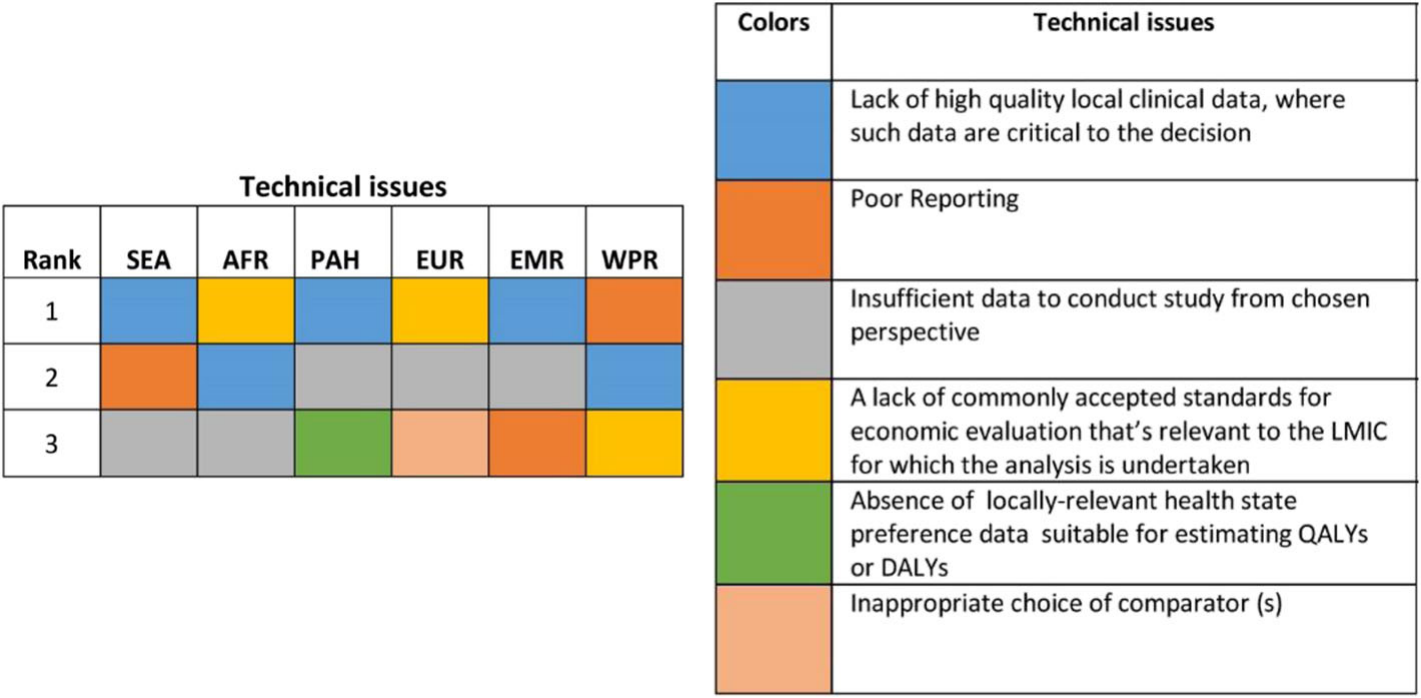
Priority technical issues in different WHO regions. *SEA* South East Asia region, *AFR* African region, *PAH* Pan American region, *EUR* European region, *EMR* Eastern Mediterranean region, *WPR* Western Pacific region

Considering each WHO region separately, the lack of high-quality local clinical data was noted as the most important issue for South East Asia (SEA), Pan America (PAH), and Eastern Mediterranean (EMR) regions, and the second most important issue for the African region (AFR). In addition to this, insufficient data to conduct the study from the chosen perspective was also noted for almost all regions except for the Western Pacific region (WPR). Poor reporting was noted for SEA, EMR and WPR as the second, third, and most important issue, respectively. Lack of commonly accepted standards was noted as being very important for AFR and the European region (EUR), while the absence of relevant health-state preference data was ranked highly only for PAH and inappropriate choice of comparator was ranked highly only for EUR (Fig. 1).

### Context-specific issues

#### Priority context-specific issues

The context-specific issues that were considered important were economic evaluations not included in the decision-making process; limited local capacity to conduct the research; and lack of funding. For scores and frequencies, please see Table 5.

**Table 5:** Ranked context-specific issues

Rank	Context-specific issue	Score	Frequency in first rank
1	Economic evaluations not included as a part of the decision-making process	26	39%
2	Limited local capacity to conduct or contextualise research	19	29%
3	Lack of funding for the necessary research	10	15%^a^
4	Misunderstandings and communications weaknesses between researchers, academia and end-users of the evidence	10	15%
5	Absence of local journal with a high-quality reviewing processes	1	2%

^a^The rank takes account of frequencies in the regional analysis

Considering each WHO region separately, exclusion of economic evaluation from the decision-making process was a priority issue for all settings, particularly in SEA, AFR, EUR and EMR. It was the second most important issue in WPR and the third in PAH. The lack of funding for research was considered high priority in most regions except EMR. It was considered the third most important issue in SEA, AFR, EUR and WPR, and the most important issue in PAH. The third most common issue was the limited capacity, which was an issue in AFR, PAH, EMR and WPR. Finally, misunderstandings and weaknesses in communication between researchers and relevant stakeholders was cited as another important challenge.

#### Context-specific versus technical issues

**Fig. 2:**
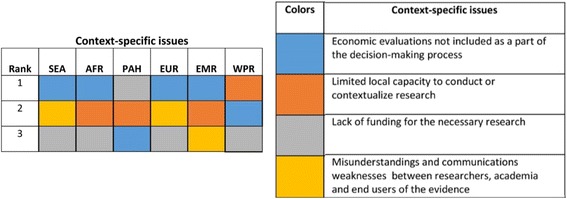
Priority context-specific issues in different WHO regions. *SEA* South East Asia region, *AFR* African region, *PAH* Pan American region, *EUR* European region, *EMR* Eastern Mediterranean region, *WPR* Western Pacific region

Sixty-six percent of respondents reported context-specific issues as more of an impediment to economic evaluation compared to technical issues (34%) (Fig. 2).

#### Proposed solutions for technical issues

Respondents’ proposed solutions for technical issues are shown in Table 6. The solutions that were most frequently proposed were the development of acceptable (i.e. understandable and perceived useful) methodological reporting guidelines for decision-makers and stakeholders in the country (*n* = 19); construction of a database for information essential for the conduct of economic evaluation (*n* = 7); and capacity-building (*n* = 7). The majority of respondents did not propose solutions.

**Table 6:** Solutions proposed by respondent and their frequency of being proposed

Proposed solution	Frequency
Development of tools and standards	
Development of standard methodological and reporting guidelines that are acceptable (i.e. understandable and perceived useful) for decision-makers and stakeholders in the country	19
ᅟImprovement or development of tools for utility weights	3
Development of standard approaches for costing	1
Generation of data and construction of databases	
Construction of a database to collate all essential data and information needed in the analysis, i.e. cost and clinical data, from data that already exists in the health systems	7
Generation of cost data through studies or standard cost lists	2
Development of utility or disutility weights for LMICs locally and/or by region	2
Conduct of studies on utility weights in the context	2
Encouragement to the conduct of local clinical studies	1
Conduct of research to estimate cost-effectiveness threshold	1
Capacity-building	
Capacity-building to increase both the quantity of economic evaluation practitioners and the quality of their work	7
Networking and system to support and appraise	
Create local system, e.g. technical committee, to support and appraise the conduct of economic evaluation	4
Engagement of relevant stakeholders in the conduct of studies and facilitating more interaction between and among different stakeholders to create buy-in	3
Creation of linkage and network with other researchers working in developing countries for knowledge and research sharing	1

## Discussion

Across all settings, the top priority technical issues were the lack of quality local clinical data, poor reporting, insufficient data to conduct the analysis from the chosen perspective, i.e. the lack of cost data, lack of commonly accepted standards for economic evaluation, and lack of local health state preference data for estimating QALYs and DALYs, respectively.

The fact that the lack of quality local clinical data ranked as high priority in the survey is not surprising. Clinical data comprises two main components, namely treatment effect, i.e. efficacy and effectiveness, and baseline risk. It is likely that data on both components are missing in LMICs. The gold standard for deriving treatment effect is randomised controlled trials (RCTs), of which the conduct is capacity, time and resource intensive such that many LMICs cannot afford to conduct them. Although data might be available through other types of research, e.g. observational study, researchers may find the quality of data derived as inadequate. Since treatment effect is transferable [9] across different contexts, researchers may borrow them; however, it is evident that many researchers (and the policy-makers they work with) doubt this either due to lack of awareness on the transferability quality of the data or the belief that it is inappropriate. The latter may be valid and can be a substantial consideration in some cases since clinical practice guidelines for some diseases in LMICs may differ significantly from those of HICs, where the treatment effect data from RCTs is richer, making it unfeasible to apply the information from those contexts. On the other hand, baseline risk data is less transferable since it is largely context specific [9], so the quality data should be gathered locally. In many LMICs, studies that focus on the baseline risk data, such as prevalence and incidence of specific diseases, can be employed. However, these studies mostly derive results from an individual health facility or in limited geographical areas, and more importantly, the quality of methodology of deriving the information can be questionable. In addition, in some diseases, there is no baseline risk data available at all; researchers may have no choice but to opt for the lowest quality data, i.e. expert opinion [5, 33].

To overcome the lack of quality local clinical data, economic evaluation practitioners demand easy-to-apply research standards that are not resource-intensive and the construction of a database, preferably at national level, for baseline risk data. With resource constraints in LMICs, the gold standard study design in HICs might not be applicable. Research methods for clinical trials and data collection that are applicable globally (or specific to LMICs) are therefore warranted to overcome the issue in deriving treatment effects. For example, instead of traditional RCTs, a pragmatic trial design that observes treatment effect of the intervention of interest in its routine clinical settings has a good potential as a solution to the measurement of treatment effect in LMICs. In addition, other types of implementation research [34] of which the validity and unbiasedness are comparable to RCTs may be developed. These would help reduce the cost of achieving clinical data that is suitable for the setting. Regarding the baseline risk data, although the construction of a national database can be resource intensive, it will benefit not only the conduct of economic evaluation but also the health system as a whole since this dataset can also be employed for other research for development in the country. This database should also include the recommendations on a tool or standard methodology for baseline risk data collection and analysis in order to ensure that high-quality data is derived and the methodology is standardised.

The lack of sufficient cost data for the chosen perspective, which is the third priority issue, is also prominent since this information is highly context specific. With the lack of cost data, researchers have to either retrieve cost data through a review of other existing related studies, if available, or collect primary data [6]. The use of secondary data from other literature can be a good solution, but researchers need to be sure that the quality of the cost data retrieved is satisfactory if such studies exist at all. On the other hand, primary data collection ensures data that is relevant to the context, but it also leads to an increased need of human and financial resources despite the constraints in LMICs. Moreover, this will introduce heterogeneity in the information obtained since methods for data collection and analysis may vary, and therefore another study in the same context may not be comparable. The survey respondents proposed the construction of standard cost lists compiling the relevant reference cost items in the economic evaluation as a potential solution. Although the process of constructing the list can be complex and resource intensive, it will lead to savings on both resources and time in the long run.

Information on health state preference data is, on the other hand, more difficult to derive, especially without existing local tools such as a tariff for converting the EQ-5D questionnaire result into utility scores. The second-best approach is to employ the information available in other jurisdictions of which the determinants of quality of life are comparable to the jurisdiction of interest. However, a LMIC is likely to be comparable to another LMIC, and most of LMICs do not have this information even for the use in their respective country. To overcome this, the survey respondent pointed out that there is a need to consider the possibility and the approach on deriving a dataset at a regional or sub-regional level. Another solution could be to develop an approach to effectively translate health state preference data from one context to another by taking into account the social, economic and health system context.

The second priority issue, poor reporting, is a weakness found in many economic evaluation studies in LMICs, and is closely linked to the fourth priority issue, which is the lack of commonly accepted standards for economic evaluation. Not only does poor reporting hamper the usability of the study since evidence-users cannot determine whether the studies are context relevant or comparable with other studies, it also prevents replication and reproduction, which are the basis for further capacity-building in related settings. Commonly accepted standards in reporting should be made available in the local context to improve this issue.

The fact that the lack of locally accepted standards was ranked fourth despite the availability of several standard guidelines and reference cases for LMICs may imply that the standards available are not perceived as relevant to the context, researchers are not well-informed of the standards’ existence, or there simply has never been a discussion to reach a consensus among researchers and policy-makers in the given contexts. As a result, researchers individually choose from a broad range of guidelines available in international for a, decide which guidelines to apply to their studies or probably do not refer to any at all. This leads to variation in the methodology and quality of studies. Therefore, reconsideration of the relevance and applicability of existing international methodological and reporting standards in LMIC contexts is necessary. If they are deemed applicable, the reason why researchers do not recognise their usability or cannot reach a consensus on a common standard should be explored and addressed. For example, the dissemination of the standards and guidelines existence and having a consensus on the standard for both reporting and methodology should be promoted. For example, there is a methodological reference case for economic evaluation in LMICs recently developed by the iDSI [35], the application of which is pursued through its partnerships and work with country governments to raise awareness and political buy-in.

On the other hand, these technical concerns are closely linked to the lack of research capacity, budget and supportive infrastructure, which are prioritised among the context-specific issues. With a limited number of trained personnel and insufficient budget, it is difficult to yield quality treatment effects from locally conducted clinical trials or to generate reliable baseline, cost and health-related preference datasets from local data collection. Therefore, local researchers should be incentivised and encouraged to conduct local studies at a high level of quality through, for example, capacity-building activities with assistance from international partners and forums.

Interestingly, although the lack of quality local clinical data was the first priority issue, only a minor group of respondents proposed solutions related to clinical information (*n* = 7). Instead, the focus of respondents was on the development of commonly accepted standards for economic evaluation (*n* = 19). This may partly be because most of the respondents did not specify the type of the standard proposed or whether a methodological or reporting standard is needed, and therefore it is difficult to distinguish between whether the proposal aims to solve the second priority issue (poor reporting) or the fourth priority issue (lack of commonly accepted standard). Moreover, respondents may anticipate that the guidelines will specify how to tackle their other technical issues of concern. However, if there is no methodological research performed, the specifications available in the guidelines alone may not be able to solve the issues in the long term. Another most frequent proposed solution was capacity-building to increase the number of researchers and the quality of their work (*n* = 4). It is noteworthy that the proposed solutions include various non-technical measures. This highlights the close link between technical and context-specific issues.

However, when considering context-specific issues, the lack of research capacity ranked only second, while researchers considered that the most important issue was economic evaluation not being included in the decision-making process. This confirms the importance of political buy-in that affects resources dedicated to research, e.g. the quality and quantity of human resources, budget allocated and supportive infrastructure. Moreover, political needs often generate demand for high-quality evidence that incentivises researchers to perform better to derive better impact from their work. Moreover, better attention given to economic evaluation will lead to a higher budget and resources allocation and, subsequently, enhanced infrastructure and capacity-building activities to improve research competency of local scholars, ranked as the next priority issue. Since, at their foundation, context-specific issues can be considered capacity issues, capacity-building activities should be promoted at different levels, i.e. capacity of evidence generators to produce evidence and capacity of evidence users to commission and translate evidence, capacity of regional network and funders to support the generation and use of quality data and evidence, and capacity of health systems to incorporate evidence in policy-making and to implement it. These may include activities such as training, awareness raising among stakeholders, stakeholder engagement, knowledge transfer and exchange, and support of the establishment of organisations specialised on economic evaluation [36].

The study also examined differences in important issues between the WHO regions. Priority technical issues and context-specific issues were homogeneous across regions and point to the same direction as the main rankings. For both technical and context-specific rankings, the three highest scoring priority issues in the global ranking also ranked highly by researchers working in each part of the world. However, for technical issues, the lack of commonly accepted standards for economic evaluation that are specific to LMICs were highlighted in Africa and developing countries in Europe, which may be rooted in the significant presence of donors [37] that conduct a variety of economic evaluations through various programmes with overlapping goals.

Although context-specific issues were considered the largest barrier impeding the use of economic evaluations, it is important to note that context-specific issues and technical issues are intertwined and should be improved simultaneously. This study only separated technical and context-specific issues in order to identify priority issues that could be addressed in future research within the context of the limitations of LMICs as well as the issues which need to be tackled though other approaches. Methodological research and the generation of tools and data, as mentioned above, can provide solutions to technical issues, but this is not the case for context-specific issues, which require a concerted effort of stakeholders to ensure the effective use of economic evaluation. However, supportive factors are in place. Global movements and players, such as WHO, have been paying attention to economic evaluation [38], and with universal health coverage becoming a major focus of many countries, the trends of demand and buy-in for economic evidence as a tool to achieve efficient and sustainable health systems are positive.

These findings are promising; however, this study is not without limitations, primarily the low response rate from certain regions. Despite sending the results to various networks, a lack of access to email databases meant that the survey reminder and compliance relied on a secondary party and not the researchers. This also affected the response rate since duplications could not be removed. The study has considerably more respondents from the SEA and WPR regions because the researchers have access to these network databases. In addition, there is no network for potential respondents in the EMR, which resulted in this region having the lowest number of respondents. The survey provided a list of issues for the respondents to choose from. Although the respondents could nominate and rank additional issues that they deemed important, there remains a potential bias since some major issues might not have been included in the list and respondents may have been less likely to consider issues that were not provided. Since the original objective of this study was to explore technical issues and their potential solution, if available, and context-specific issues were added to ensure comprehensiveness of the survey, respondents were asked to propose solutions only for technical issues and not for context-specific issues. Finally, the review of literature discussing methodological issues included both LMICs and HICs and the framework for reviewing these economic evaluations in LMICs was obtained from the CRD, which is based on HIC methods and experts. Thus, the issues identified are not exclusive to the LMIC context. Further studies on the methodological issues in LMICs may be needed, for example, to identify methodological issues by first conducting an interview with scholars in LMICs to obtain a better understanding of their methodological problems and then constructing a survey based on the findings.

## Conclusions

There are many priority issues which could – and should – be solved through methodological research to find an appropriate approach for use in LMICs and improve the standard of economic evaluation; on the other hand, in terms of non-technical issues, many issues also need to be solved through capacity-building, etc. Concerted efforts are therefore needed not only among international donors and health initiatives but also among governments and local entities in the respective countries in order to overcome these problems and strengthen the use of economic evaluation.

The results of this study provide a preliminary understanding of the issues faced by researchers in developing countries. The most important technical issues were the lack of quality local clinical data, poor reporting, insufficient data to conduct the analysis from the chosen perspective (i.e. the lack of cost data), lack of context-relevant standards for economic evaluation, and lack of local health-related preference data, respectively. The most important context-specific challenge was that economic evaluation is not included in the decision-making process. Context-specific issues are also considered to be a bigger challenge to the conduct of quality economic evaluations as compared to purely technical or methodological issues.

The results will be inputted into the iDSI programme for future work on methodological development in 2016–2019, with the aim to promote a generation of robust evidence for health resource allocation to policy-makers. Based on findings from this study, an online database is being created as a comprehensive web-based knowledge-sharing platform that addresses methodological issues regarding policy-relevant research. An online resource, entitled ‘Guide to health Economic Analysis and Research’ can be accessed at the www.gear4health.com. It will showcase the results of this research as well as providing quick consultation to economic practitioners who encounter methodological difficulties in the conduct of studies.

## Additional files

Additional file 1: Issues included in the questionnaire. (DOCX 14 kb)

Additional file 2: Further information of respondents. (DOCX 16 kb)

## Abbreviations

AFR: WHO African region
CRD: Centre for Reviews and Dissemination
DALYs: disability-adjusted life years
EMR: WHO Eastern Mediterranean region
EUR: WHO European region
HTA: health technology assessment
iDSI: International Decision Support Initiative
LMICs: low- and middle-income countries
NHS EED: National Health System Economic Evaluation Database
PAH: WHO region of the Americas
QALYs: quality-adjusted life years
RCT: randomised controlled trial
SEA: WHO South-East Asia region
WPR: WHO Western Pacific region
