# Supplementary material for: Identifying priority technical and context-specific issues in improving the conduct, reporting and use of health economic evaluation in low- and middle-income countries
Source: Health Res Policy Syst. 2018 Feb 5;16:4. doi: 10.1186/s12961-018-0280-6 (PMC5800077; doi:10.1186/s12961-018-0280-6)
Supplement: Supplementary file 1 — Issues included in the questionnaire. (DOCX 14 kb) [file 12961_2018_280_MOESM1_ESM.docx]

## **S1 Issues included in the questionnaire**

Technical issues

|  | **Issues** |
| --- | --- |
| A | Poor Reporting, e.g.   - Perspective of analysis not stated - Lack of details of the target population or sub-populations relating to the interventions being evaluated - Lack of detail on the methods used to derive the measure of benefit - Lack of details to enable checking of data sources for benefits / effectiveness - Lack of details to enable checking of data sources for costs - No details of the price year used as the basis of the cost analysis - Methodology not presented in a clear and reproducible manner |
| B | A lack of commonly accepted standards for economic evaluation that’s relevant to the LMIC for which the analysis is undertaken |
|  | **Benefits / Effectiveness** |
| C | Lack of high quality local clinical data, where such data are critical to the decision |
| D | Clinical data not based on systematic review; or primary clinical data not compared with similar studies done elsewhere |
| E | Absence of locally-relevant health state preference data suitable for estimating QALYs or DALYs |
|  | **Costs** |
| F | Insufficient data to conduct study from chosen perspective |
| G | Uncritical use of charges for cost data |
|  | **Analysis** |
| H | Inappropriate choice of comparator (s) |
| I | Time horizon too short to capture relevant costs and health effects |
| J | All impacts implied by the chosen perspective not investigated |
| K | Unexplained differences in discount rates; or the discount rate used does not reflect reality in the local context |
| L | No incremental analysis |
| M | No, or inappropriate, sensitivity analysis |
| N | No budget constraints or thresholds considered |
| O | Equity and/or gender implications not considered |
| P | Generalizability not discussed |

Context-specific issues

|  | **Issues** |
| --- | --- |
| A | Economic evaluations not included as a part of the decision-making process |
| B | Lack of funding for the necessary research |
| C | Limited local capacity to conduct or contextualize research |
| D | Absence of local journal with a high quality reviewing processes |
| E | Misunderstandings and communications weaknesses between researchers, academia and end users of the evidence |
