# Supplementary material for: Identifying priority technical and context-specific issues in improving the conduct, reporting and use of health economic evaluation in low- and middle-income countries
Source: Health Res Policy Syst. 2018 Feb 5;16:4. doi: 10.1186/s12961-018-0280-6 (PMC5800077; doi:10.1186/s12961-018-0280-6)
Supplement: Supplementary file 2 — Further information of respondents. (DOCX 16 kb) [file 12961_2018_280_MOESM2_ESM.docx]

## S2 Further information of respondents

Most respondents work in their home regions, although some work in regions other than theirs. For example, many respondents from PAH (14 respondents) reported working in SEA (8 respondents), AFR (7 respondents), EUR (5 respondents), and the WPR (6 respondents) as well. Respondents from EUR (12 respondents) also work in SEA (5 respondents) and AFR (12 respondents). Similarly, some respondents from WPR (23 respondents) also work in SEA (6 respondents).

Table 1 Respondents from other regions working outside their own

| **Regions of work**  **Home Regions** | **SEA** | **AFR** | **PAH** | **EUR** | **EMR** | **WPR** |
| --- | --- | --- | --- | --- | --- | --- |
| Southeast Asia | 23 | 1 | 1 |  |  | 1 |
| Africa |  | 14 |  | 1 |  |  |
| America | 8 | 7 | 14 | 5 | 2 | 6 |
| Europe | 5 | 12 | 2 | 12 | 2 | 3 |
| Eastern Mediterranean |  |  |  |  | 2 |  |
| Western Pacific | 6 |  |  |  |  | 23 |

A number of other analyses were conducted to further outline the characteristics of the survey respondents. The highest number of respondents was from Thailand, then the United Kingdom, Malaysia, the United States, and Brazil, and China.

Table 2 Number of Country Respondents by rank

| Ranking (no.) | Country | **Response Count** |
| --- | --- | --- |
| 1 | TH - Thailand | 15 |
| 2 | GB - United Kingdom | 13 |
| 3 | MY - Malaysia | 10 |
| 4 | US - United States | 10 |
| 5 | BR - Brazil | 6 |
| 6 | CN - China | 6 |
| 7 | PH - Philippines | 5 |
| 8 | IN - India | 4 |
| 9 | ZA - South Africa | 4 |
| 10 | ID - Indonesia | 3 |
